# Laparoscopic resection for appendiceal mucocele secondary to endometriosis: A case report

**DOI:** 10.1097/MD.0000000000036277

**Published:** 2023-11-24

**Authors:** Hitoshi Hara, Seito Shimizu, Yasuhide Muto, Tomoki Kido, Ryohei Miyata

**Affiliations:** a Department of Surgery, Social Welfare Organization Saiseikai Imperial Gift Foundation Inc., Saiseikai Kazo Hospital, Kazo, Saitama, Japan.

**Keywords:** appendiceal endometriosis, case report, laparoscopic surgery, mucocele of the appendix

## Abstract

**Introduction:**

This case report describes a patient who underwent laparoscopic resection of the mucocele of the appendix secondary to endometriosis, a rarity in clinical practice.

**Patient concerns:**

The patient was a 38-year-old woman with a history of endometriosis and an ovarian cyst who sought medical advice with a chief complaint of mild right lower abdominal pain.

**Diagnoses:**

Computed tomography and ultrasonography of the abdomen revealed a cystic lesion at the distal end of the appendix without definitive findings of malignancy. Colonoscopy revealed a submucosal tumor-like elevation at the appendiceal orifice. Appendiceal mucocele was suspected preoperatively.

**Interventions:**

The lesion was resected laparoscopically. Secondary ileocecal resection with lymphadenectomy was possible if the resected specimen was pathologically diagnosed as a malignant tumor with the risk of lymph node metastasis.

**Outcomes:**

The resected specimen was pathologically diagnosed as an appendiceal mucocele secondary to endometriosis; therefore, additional surgery was avoided.

**Conclusion:**

Although appendiceal mucoceles secondary to endometriosis are rare, laparoscopic surgery in which only the lesion was resected is a useful strategy for the treatment and pathological diagnosis of appendiceal mucoceles without findings of malignancy.

## 1. Introduction

Mucocele of the appendix is a rare disease characterized by partial or complete distension of the appendix due to some obstruction or stenosis. This disease is observed in 0.08% to 0.33% of appendectomies.^[1,2]^ The causes of obstruction and stenosis are classified histologically into nonneoplastic and neoplastic.^[3]^ Neoplastic luminal strictures include benign and malignant tumors, and the rupture of these mucoceles sometimes produces pseudomyxoma peritonei.^[3]^ Endometriosis (0.05%–0.15% of appendectomies) is one of the rare causes of appendiceal mucocele because of nonneoplastic luminal strictures.^[1,2,4]^ Although the mucocele of the appendix requires surgery, ileocecal resection with lymphadenectomy is often performed by laparotomy because of the concern for rupture and the risk of malignant tumors. Mucocele of the appendix secondary to endometriosis is rare, but we found that laparoscopic surgery for resecting only the lesion is a useful strategy for treatment and pathological diagnosis in cases without malignant findings. Our case report is the first to describe the pathological findings of an appendiceal mucocele caused by crypt gland obstruction resulting from fibrosis of endometriosis. Our study also describes laparoscopic surgery as a beneficial modality for treating mucocele of the appendix secondary to endometriosis.

## 2. Case presentation

### 2.1. Patient information, clinical findings, timeline, and diagnostic assessment

**Figure 1.**
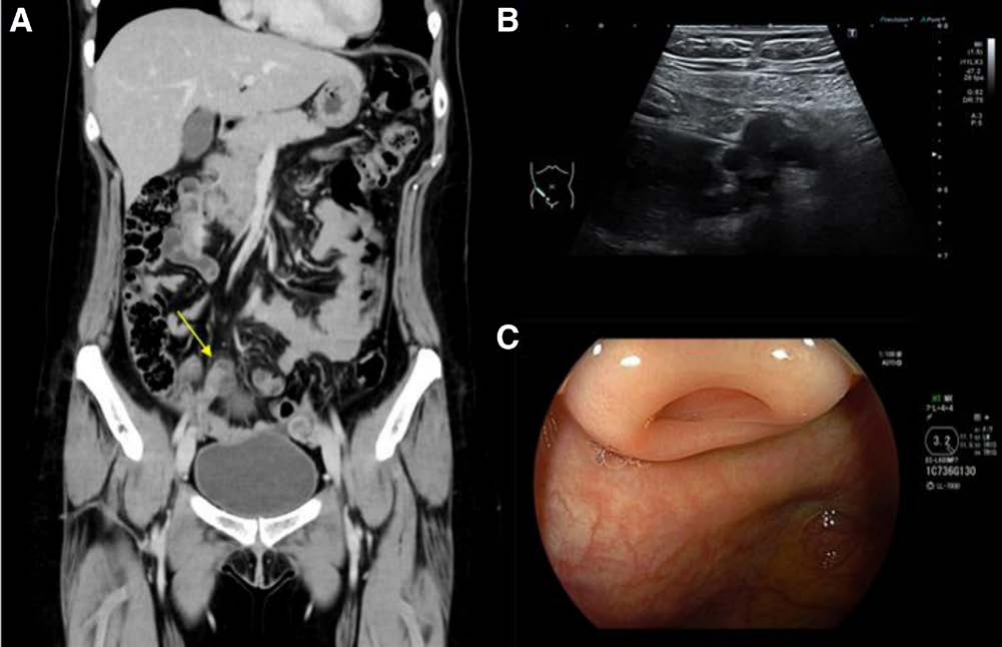
Lesion visualization. (A) Computed tomography (yellow arrow) and (B) ultrasonography of the abdomen showing a cystic lesion at the appendiceal distal end. (C) Colonoscopy showing a submucosal tumor-like elevation at the appendiceal orifice.

A 38-year-old woman with a history of endometriosis and an ovarian cyst sought medical advice with a chief complaint of right lower abdominal pain. On physical examination, the patient presented with no fever and mild right lower abdominal tenderness without rigidity. Blood tests revealed no evidence of inflammation. Computed tomography and ultrasonography of the abdomen revealed a cystic mass at the distal end of the appendix without definitive findings of malignancy (Fig. 1A and B). Colonoscopy revealed submucosal tumor-like elevation at the appendiceal orifice (Fig. 1C). Consequently, appendiceal mucocele was suspected preoperatively. Although the patient had a history of endometriosis, hormonal therapy was not performed to improve the chance of pregnancy. Cancer antigen 125 level, often elevated in endometriosis patients, was not high in this case.

### 2.2. Therapeutic intervention

**Figure 2.**
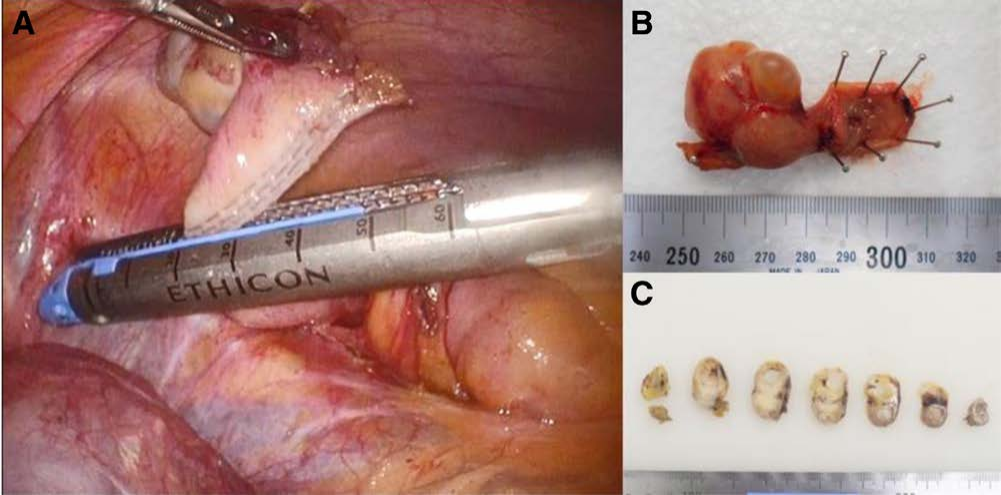
Surgical findings. (A) Laparoscopic partial resection of the cecum was performed to resect the lesion completely. (B and C) Macroscopic appearance of the resected specimen showing an irregular thin-walled cystic mass at the distal end of the appendix.

We performed a partial laparoscopic resection of the cecum to resect the lesion completely (Fig. 2A). We were aware of the need for secondary ileocecal resection with lymphadenectomy if the specimen was pathologically diagnosed as a malignant tumor with possible lymph node metastases. During surgery, adhesions in the pelvis and ovarian cysts due to endometriosis were observed; however, findings of appendiceal mucocele rupture and definitive findings of malignancy were not observed.

### 2.3. Follow-up and outcomes

**Figure 3.**
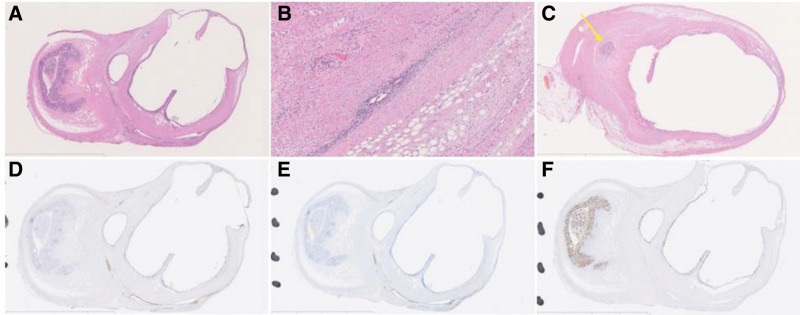
Histopathological findings. (A) Pathological findings showed an irregular unilocular mucinous retention cyst. The cystic wall was lined with columnar epithelium with a thin muscular layer with endometriosis lesions and fibrosis. (B) Endometriosis was observed in the muscle layer. (C) The mucus-filled cyst arose from the deep mucosal area near the tip of the appendix. The non-expanded tip of the appendix (yellow arrow) revealed that obstruction of the luminal structure of the vermiform appendix was not present. Immunostaining showed (D) CD10-positive and (E) ER-positive staining for endometriosis and (F) CDX2-positive staining for colonic epithelium. (A, C–F: 4×, B: 200× magnification) (A–C: hematoxylin & eosin staining).

The resected specimen macroscopically showed the cystic lesion at the top of the appendix (Fig. 2B). The cystic lesion was an irregular unilocular cyst containing mucus (Fig. 2C) and was pathologically diagnosed as endometriosis with a retention cyst (Fig. 3A–F). The cyst wall was lined with columnar epithelium without atypia, which was CDX2-positive for immunostaining. The cyst was surrounded by a thin, smooth muscle layer with CD10- and ER-positive endometriotic lesions and fibrosis and continued from the deep mucosal area near the tip of the appendix. No obstruction was observed in the luminal structure of the appendix from the root to the tip. We concluded that this mucus-retaining cyst was caused by the obstruction of a crypt gland of the appendiceal mucosa due to fibrosis associated with endometriosis.

The postoperative course was uneventful, and the patient was discharged on postoperative day 4. Because endometriosis is a benign disease, additional surgery and follow-up were not required. After the surgery, the right lower abdominal pain disappeared.

## 3. Discussion

In this study, we described a rare case of appendiceal mucocele secondary to endometriosis. Because preoperative examinations and surgical findings did not reveal findings suggestive of malignant disease and rupture, we performed laparoscopic surgery to remove the lesion completely as a partial resection of the cecum. As a result, a rare condition of appendiceal mucocele secondary to endometriosis was diagnosed pathologically, and additional surgery was unnecessary.

The appendiceal mucocele is macroscopic in appearance and presents as a cystic lesion where the appendix is partially or completely expanded.^[5]^ Some pathological entities, including benign and malignant diseases, cause appendiceal mucocele.^[5]^ Diagnosing benign or malignant appendiceal lesions preoperatively is challenging for anatomical reasons, except in cases with findings suggestive of malignant diseases such as metastasis or pseudomyxoma peritonei.^[4]^ Surgery is required to treat appendiceal mucocele, but the optimal surgical procedure differs depending on the causative disease. For benign diseases, resection of the lesion alone is sufficient. Although malignant, low-grade appendiceal mucinous neoplasms and T1a appendiceal cancer do not cause lymph node metastasis, thus resection of the lesion alone without lymphadenectomy can serve as sufficient treatment.^[3]^ Ileocecal resection with lymphadenectomy is required for more advanced appendiceal cancers than T1a. However, in clinical practice, if benign or malignant lesions are not diagnosed preoperatively, ileocecal resection with lymphadenectomy is often selected because of the risk of malignant diseases. This indicates that more invasive surgery has been performed in benign diseases or malignant tumors without the possibility of lymph node metastasis. Our study found that a pathological diagnosis using permanent specimens from the lesion resection can avoid unnecessary and invasive surgery.

In our case, we could not make a preoperative histological diagnosis, but there were no findings suggestive of malignant disease, such as metastasis or pseudomyxoma peritonei. Therefore, we considered certain diseases, including appendiceal endometriosis, for which surgery can be performed by completely removing the lesion. Laparoscopic surgery is minimally invasive and esthetically superior and has the advantage of less adhesion when additional surgery is required. A pathological diagnosis of appendiceal endometriosis was made based on the laparoscopic surgery specimen, and excessive secondary surgery was unnecessary. Except for cases in which a malignant tumor could be diagnosed preoperatively, the first laparoscopic resection of the lesion alone is useful, with the option for a secondary surgery depending on the results of the histological diagnosis.

Although endometriosis of the appendix is rare, unique pathological findings were observed in our case. Most appendiceal mucoceles are caused by epithelial lesions associated with mucin production, such as hyperplastic polyps, adenomas, and carcinomas.^[5]^ A minority of appendiceal mucoceles are caused by retention of mucin secondary to obstruction or stenosis of the appendix due to fecaliths, post-inflammatory scarring, and rarely are they caused by endometriosis (called obstructive, retention, and simple mucoceles).^[5]^ Endometriosis is a common disease in women, defined as functioning endometrial tissue in a location outside of the uterus. Small cystic lesions called blueberry spots, in which menstrual blood accumulates, are often observed with endometriotic lesions, although there were no blueberry spots in our case. The pathological findings confirmed the relationship between the columnar epithelium and the endometriotic lesions using CD10, ER, and CDX2. Such histopathological diagnostic methods using immunostaining are useful in distinguishing endometriotic lesions from the columnar epithelium of the appendix and revealed findings of a rare mucocele of the appendix^[6]^ caused by a crypt gland obstruction of the appendiceal mucosa. Klingbeil et al^[7]^ reported a case where an appendiceal mucocele consisted of endometriosis and low-grade appendiceal mucinous neoplasms; therefore, our pathological findings may have had the potential of developing malignancy in the future.

The obstructive mucocele of the appendix secondary to endometriosis is rare, and reported cases with a similar condition were previously described in only 8 cases.^[5,8–13]^ In these cases, endometriosis was the preoperative diagnosis in only 1 case in which endometriosis was diagnosed by previous laparoscopy.^[9]^ In these cases, appendiceal mucocele was caused by obstruction of the lumen of the appendix (5 cases), obstruction of the appendiceal lumen and the crypt gland of the mucosa (1 case), and appendiceal mucocele with an indeterminate cause (2 cases). Ileocecal resection was performed in 1 case, right hemicolectomy in 1 case with rupture, hysterectomy with appendectomy in 1 case with surgery for endometriosis, and lesion resection alone as appendectomy or partial resection of the cecum in 5 cases. Except for cases of rupture of the appendiceal mucocele and surgery for endometriosis, ileocecal resection was performed in 1 of 6 cases. None of the patients underwent laparoscopic surgery. To the best of our knowledge, our case report is the first to describe the pathological findings of an appendiceal mucocele caused by crypt gland obstruction resulting from fibrosis of endometriosis. Our study also describes laparoscopic surgery as a beneficial modality for treating mucocele of the appendix secondary to endometriosis.

The case report has several limitations. En bloc resection cannot be achieved when 2 stages of extensive surgery are required in cases of malignancy. Therefore, if preoperative or intraoperative findings are positive for malignant tumors, such as metastasis or pseudomyxoma peritonei, ileocecal resection with lymphadenectomy should be selected. Because open surgery rather than laparoscopic surgery reduces the chances for intraoperative rupture of the mucocele of the appendix, careful surgical skills are required in laparoscopic surgery.

## 4. Conclusions

Mucoceles of the appendix secondary to endometriosis are rare and difficult to diagnose preoperatively. In cases of mucocele of the appendix without definitive findings of malignancy, complete laparoscopic resection of only the lesion is a useful strategy for treatment and pathological diagnosis.

## Acknowledgments

The authors thank Dr. Hiroko Ogata for her support in pathological diagnosis, Editage (www.editage.jp) for English language editing, and Ms. Satoko Fukaya who works at our hospital library.

## Author contributions

**Conceptualization:** Hitoshi Hara.

**Data curation:** Hitoshi Hara, Seito Shimizu, Yasuhide Muto, Tomoki Kido, Ryohei Miyata.

**Investigation:** Hitoshi Hara.

**Resources:** Hitoshi Hara, Ryohei Miyata.

**Validation:** Hitoshi Hara.

**Visualization:** Hitoshi Hara.

**Writing – original draft:** Hitoshi Hara.

**Writing – review & editing:** Hitoshi Hara, Ryohei Miyata.
